# SARS–associated Coronavirus Replication in Cell Lines

**DOI:** 10.3201/eid1201.050496

**Published:** 2006-01

**Authors:** Matthew Kaye, Julian Druce, Thomas Tran, Renata Kostecki, Doris Chibo, Jessica Morris, Mike Catton, Chris Birch

**Affiliations:** * Victorian Infectious Diseases Reference Laboratory, North Melbourne, Victoria, Australia

**Keywords:** SARS-CoV, cell susceptibility, cytopathic effects, research

## Abstract

Given the potential for laboratory-associated severe acute respiratory syndrome–associated coronavirus (SARS-CoV) infections, we must know which cell lines are susceptible to the virus. We investigated 21 cell lines routinely used for virus isolation or research. After infection with SARS-CoV, cells were observed for cytopathic effects, and quantitative real-time polymerase chain reaction was used to measure ongoing viral replication. An indirect immunofluorescence assay was also used as a confirmatory test. The study identified 10 new cell lines capable of supporting the replication of SARS-CoV and confirmed the susceptibility of 4 cell lines previously reported. This study shows that SARS-CoV can be isolated in several cell lines commonly used for diagnostic or research purposes. It also shows that SARS-CoV can achieve high titers in several cell lines, sometimes in the absence of specific cytopathic effects.

## Abstract 2

Virus can replicate in several common cell lines, sometimes without cytopathic effect.

Severe acute respiratory syndrome (SARS) was first observed in 2002 when cases of a life-threatening atypical pneumonia occurred in Guangdong Province, China (*1*). A novel coronavirus (CoV), designated SARS-CoV, was quickly identified as the etiologic agent (*1**,**2*). Although the origins of the virus have not been established, evidence suggests that it is an animal virus that was recently transmitted to humans (*3*). Several wildlife species consumed as delicacies in southern China, including Himalayan masked palm civets, Chinese ferret badgers, and raccoon dogs, possess antibodies consistent with natural infection with related CoVs (*4*).

Unlike the other currently recognized human CoVs, HCoV-229E, HCoV-OC43, HCoV-NL63, and HKU1, which usually cause mild upper respiratory tract infections and occasionally pneumonia in older adults, neonates, and immunocompromised patients (*5**–**8*), SARS-CoV causes severe febrile lower respiratory tract illness that leads to pneumonia and acute respiratory distress (*9**,**10*). Death from progressive respiratory failure due to alveolar damage occurs in ≈10% of patients with symptomatic infection (*2**,**10*). Currently the world is free of SARS, but we cannot predict whether the virus will reemerge. The most probable sources of future infections are exposure to animal reservoirs or laboratories where SARS-CoV is manipulated for research purposes. Indeed, since the first epidemic, SARS has occurred on 3 occasions as a result of breaches in laboratory biosafety procedures (*11**–**13*). This finding highlights the importance of safely handling SARS-CoV, especially in diagnostic virology laboratories where virus isolation is performed and in research laboratories where infectious virus is handled.

SARS-CoV was first isolated in Vero E6 and FRhK cells injected with clinical specimens as part of early attempts to identify the etiologic agent of SARS (*10**,**14*). Simultaneously, these investigations showed that SARS-CoV could not replicate in a number of other cell lines routinely used for respiratory virus isolation. More recently, additional human and animal cell lines that support SARS-CoV replication have been identified (*15*). Given the potential for SARS-CoV infection to occur in a laboratory setting, we must be aware of cell lines in which it can replicate. Therefore, we investigated the susceptibility of a number of cell lines to SARS-CoV. These cells were derived from a variety of species and tissues and included those capable of supporting the replication of respiratory and enteric viruses.

## Materials and Methods

### Virus

An isolate of SARS-CoV, strain HKU 39849, was passaged on 2 occasions in Vero E6 cells to establish a high-titer stock that was used in all infectivity experiments. Because SARS-CoV is classified as a risk group level 4 pathogen in Australia, all procedures performed with the virus, including infecting cell lines and viral lysis before RNA extraction, were carried out in a physical containment level 4 (PC4) laboratory.

### Cell Lines

The cell lines investigated for their susceptibility to SARS-CoV are shown in the Table. They were chosen because they were present in our cell repository and were used either routinely or occasionally for virus isolation attempts as part of diagnostic or research projects. Confluent cells were maintained at 34°C in 25-mL flasks (Nunc, Roskilde, Denmark) containing 10 mL appropriate maintenance medium supplemented with fetal bovine serum (FBS) (Thermo Trace, Melbourne, Victoria, Australia), 100 U/mL penicillin, and 100 μg/mL streptomycin (JRH Biosciences, Lenexa, KS, USA). BGM, FRhK, HEK-293, HEL, Hep G2, L20, MA-104, pCMK, and RD-A cell lines were all maintained in modified Eagle medium (MEM) supplemented with 10% FBS. MDCK cells were maintained in MEM supplemented with 5% FBS. HeLa-T cells were maintained in basal medium Eagle supplemented with 10% FBS. COS, Huh-7, Vero, and Vero E6 cell lines were maintained in Dulbecco modified Eagle medium (DMEM) supplemented with 10% FBS. CV-1, Hep-2, LLC-Mk2, MEK, and RK-13 cells were maintained in 199 medium with 5% FBS, and A549 cells were maintained in RPMI 1640 medium supplemented with 10% FBS. Confluent cells were infected with SARS-CoV, which resulted in a multiplicity of infection of 1.7 (results not shown) or were mock-infected with medium only. An additional flask was also prepared in which the original inoculum was incubated under the same experimental conditions but within a cell-free environment.

**Table:** Susceptibility of cells to SARS-CoV*

Cell line	Species of origin	Cell type	CPE	IDFA	Quantitative PCR Ct (days 4, 7, and 11)
Supports replication					
BGM	Monkey, buffalo green	Kidney epithelium	+	+	15, 15, 14
COS	Monkey	Derivative of CV-1	–	+	31, 33, 32
CV-1	Monkey, African green	Kidney fibroblast	+	+	14, 15, 15
FRhK	Monkey, rhesus	Fetal kidney	+	+	16, 16, 15
LLC-Mk2	Monkey, rhesus	Kidney epithelium	+	+	15, 14, 14
MA-104	Monkey, African green	Kidney epithelium	+	+	17, 15, 15
MEK	Monkey	Embryonic kidney	–	+	19, NT, 16
pCMK†	Monkey, cynomolgus	Primary kidney	+	+	20, 18, 17
Vero	Monkey, African green	Kidney epithelium	+	+	14, NT, NT
Vero E6†	Monkey, African green	Clone of Vero	+	+	14, NT, NT
HEK-293†	Human	Fetal kidney	+	+	16, 16, 17
Hep G2	Human	Liver hepatocellular carcinoma	+	+	23, 23, 20
Huh-7†	Human	Liver hepatocellular carcinoma	+	+	15, 15, 16
RK-13	Rabbit	Kidney epithelium	+	+	19, 17, 21
Does not support replication					
A549†	Human	Lung carcinoma epithelium	–	–	33, 32, 35
HEL†	Human	Diploid fetal lung fibroblast	–	–	31, 34, 38
HeLa-T	Human	Cervical epithelium	–	–	32, 33, 36
Hep-2	Human	Epithelium derived from HeLa-T	–	–	31, 32, 36
RD-A	Human	Rhabdomyosarcoma	–	–	33, 32, 35
MDCK†	Canine	Kidney epithelium	–	–	32, 37, 41
L20	Murine	Express poliovirus receptor	–	–	33, 32, 35

*SARS-CoV, severe acute respiratory syndrome–associated coronavirus; CPE, cytopathic effect; IDFA, indirect immunofluorescence assay; PCR, polymerase chain reaction; Ct, cycle threshold; NT, not tested. †These cell lines were tested as part of another study (*14*), and the results confirmed as part of this study.

On days 4, 7, and 11 after infection, cells were observed for SARS-CoV–specific cytopathic effects (CPE), supernatants were collected for virus detection and quantification by polymerase chain reaction (PCR), and the maintenance medium was replaced. Cells were tested for virus-specific antigens with an indirect immunofluorescence assay 11 days after infection with SARS-CoV if no CPE was observed (or when CPE developed that involved at least 75% of the cell monolayer). Eleven days after infection, cell lines negative for indicators of viral replication were blind-passaged twice for 7 days by adding 100 μL culture supernatant to the cells in question as well as to the highly susceptible Vero E6 cells. During these passages, cells were observed for SARS-CoV–specific CPE, and after the second passage, supernatants were collected for virus detection and quantification by PCR.

### RNA Extraction

A 300-μL volume of lysis buffer containing guanidinium thiocyanate and Triton X-100 (Roche Diagnostics, Mannheim, Germany) was added to 200 μL supernatant from cell cultures that had either been infected with SARS-CoV or were mock-infected. These samples were removed from the PC4 laboratory to a PC2 laboratory, where they underwent nucleic acid extraction with a MagNA Pure LC Total Nucleic Acid Isolation Kit with a MagNA Pure LC automated extraction robot (Roche Diagnostics). A 10-μL volume of eluate was treated for 10 min at 65°C and added to 12 μL reverse transcription master mix containing 5.2 A_260_ U/mL random hexamers (Roche Diagnostics), 0.17 μmol/L deoxynucleoside triphosphates (Roche Diagnostics), and 7.5 U AMV-RT enzyme (Promega, Madison, WI, USA). After incubation at 42°C for 30 min, then 100°C for 10 min, cDNA products were stored at 4°C until analyzed by PCR.

### Quantitative Real-time PCR for SARS-CoV

Real-time PCR that amplified an 81-bp fragment of the nucleoprotein gene was used to detect and quantify SARS-CoV by reference to a cycle threshold (Ct). The assay used ABI-7000 Prism instrumentation (Applied Biosystems, Foster City, CA, USA) with primers and probes designed with the associated Primer Express software. The forward primer was SARNP-F: 5´-CCC AGA TGG TAC TTC TAT TAC CTA GGA-3´. The reverse primer was SARNP-R: 5´-CCA TAC GAT GCC TTC TTT GTT AG-3´. The probe was SARNP-P: 6FAM 5´-AAG CTT CAC TTC CCT ACG G-3´ with 3´ MGB. For real-time PCR, 5 μL template cDNA was added to ABI TaqMan Universal PCR Master Mix (Applied Biosystems) containing 0.9 μmol/L each primer and 0.2 μmol/L probe in a total volume of 45 μL. The cycling conditions were as follows: 2 min at 50°C, 10 min at 95°C, then 45 cycles of 15 s at 95°C and 1 min at 60°C. Reference to a standard curve (not shown) demonstrated that negative changes in Ct values of 3.6 represented increases in virus titer of 1.0 log_10_.

### Indirect Immunofluorescence Assay

Cells were collected 11 days after infection if no CPE was observed by microscopy or on the day they developed CPE involving at least 75% of the cell monolayer. Cells were manually scraped from monolayers into 1 mL culture medium, then subjected to 50 kGy gamma radiation before being spotted onto a slide, air dried, and fixed in acetone for 10 min. Earlier testing showed that this dose of gamma radiation reduced the titer of SARS-CoV by at least 10^6^ 50% tissue culture infectious doses (results not shown). A 10-μL volume of diluted convalescent-phase serum from a SARS-CoV–infected patient was added to the fixed cells followed by incubation at 37°C for 30 min in a humidified chamber. The slides were washed twice with phosphate-buffered saline (PBS), dried, and each cell spot overlaid with 10 μL anti-human fluorescein isothiocyanate–conjugated secondary antibody (BioMérieux, Durham, NC, USA) for 30 min at 37°C. The slides were washed twice with PBS before they were mounted with cover slips. Virus-specific immunofluorescence was read by using an Axioskop UV microscope (Zeiss, Oberkochen, Germany). The final results for the indirect immunofluorescence assay, as shown in the Table, were based on the observations of 2 independent readers.

## Results

Susceptibilities to SARS-CoV of the cell lines we investigated are shown in the Table. The results obtained on 21 lines are indicated: 14 were tested for the first time, and 7 had been previously reported by others (*15*). Of the 7 cell lines tested previously, we confirmed previous data that showed that 4 of them could support replication. Of the 14 lines tested for the first time, 10 were shown to support replication of SARS-CoV. In general, cells derived from nonhuman primate kidneys were susceptible. A human liver cell line (Hep G2) and rabbit kidney cells (RK-13) also supported replication.

**Figure 1:**
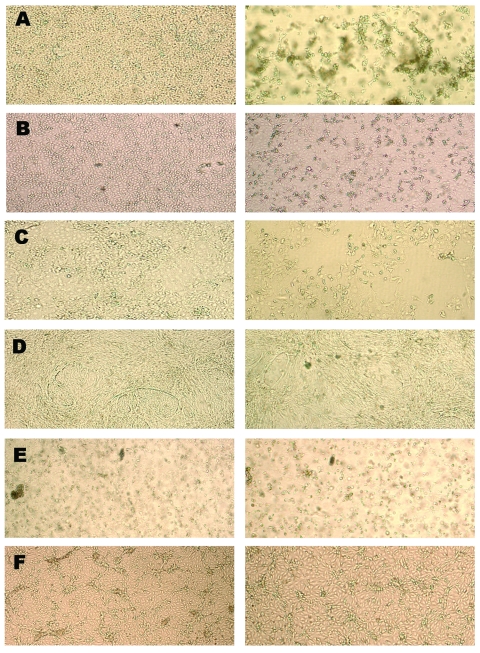
Cytopathology of uninfected cells (left column) and the same cells infected in vitro with severe acute respiratory syndrome–associated coronavirus (right column). A) Vero cells day 4 after infection. B) MA-104 cells day 4 after infection. C) Huh-7 cells day 11 after infection. D) pCMK cells day 11 after infection. E) COS cells day 11 after infection. F) MEK cells day 11 after infection.

**Figure 2:**
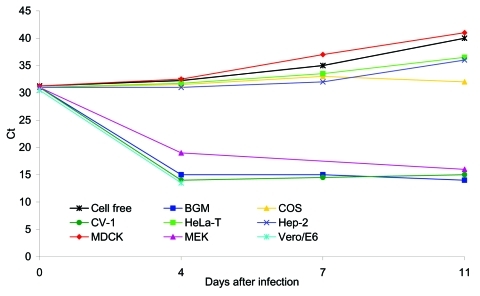
Cycle threshold (Ct) changes measured by real-time polymerase chain reaction versus days after infection of the indicated cell lines. The cell-free sample had an initial Ct of 31, which rose to 40 by day 11. Reductions in the Ct or flat-line Ct values (e.g., COS cells) indicate replication of the virus. Continued increases in Ct above the initial value of 31 by days 7 and 11 indicate failure to replicate.

SARS-CoV replication in BGM, CV-1, FRhK, LLC-Mk2, MA-104, pCMK, RK-13, and Vero cell lines produced a CPE as early as day 4 after inoculation, with evidence of high levels of virus-specific RNA established by quantitative PCR. CPE was focal, with cell rounding and a refractivity that was soon followed by cell detachment, and CPE quickly spread to involve the entire cell monolayer (Figure 1). In contrast, neither MEK nor COS cells produced a SARS-CoV–specific CPE (Figure 1), despite evidence of rapid (MEK) or limited (COS) replication, as determined by quantitative PCR (Figure 2) and indirect immunofluorescence testing (not shown). For the cell lines capable of supporting SARS-CoV replication, immunofluorescence results confirmed quantitative PCR results in all cases (Table).

Figure 2 shows the quantitative PCR results for representative cell lines for the 11 days during which isolation was attempted. The results are depicted as Ct values relative to the Ct values of a cell-free preparation. The cell-free preparation had an initial Ct value of 31, obtained when the original inoculum was seeded into a flask containing 10 mL DMEM. This input Ct increased to a Ct value of 40 by day 11 after infection. The supernatants from BGM, CV-1, MEK, Vero, and Vero E6 cell lines yielded Ct values 12–17 units lower than the initial cell-free inoculum by day 4 after infection. This number equated to titer increases 3.3–4.7 log_10_/mL above the input virus for these cells. The results for HeLa-T, Hep-2, and MDCK cells, representing cell lines that do not support SARS-CoV replication, are shown in Figure 2. In these cell lines, the Ct values at 4 days after infection were at levels similar to those of the cell-free inoculum. At later times, after successive media changes, Ct values increased in a manner similar to that of the cell-free control preparation, indicating dilution of input virus and absence of any subsequent viral replication. Blind passaging of supernatant fluid from these cell lines confirmed these results (not shown). In contrast, Ct values for COS cells did not change over the course of the experiment, which suggests that viral replication occurred at a low level, sufficient to maintain similar viral titers to those of input levels through several medium changes.

## Discussion

After the SARS epidemic ended, several cases have occurred as a direct or indirect result of breaches in laboratory biosafety (*11**–**13*). These breaches highlight the need to safely handle virus in the laboratory, which includes knowing which cell lines may be susceptible to infection. In this study we add to the list of cells known to support replication of SARS-CoV.

Our approach to establishing susceptibility to infection was to use quantitative PCR supported by immunofluorescence testing. The quantitative PCR was used to distinguish ongoing viral production from input virus. Other groups have used alternative strategies to investigate SARS-CoV replication, including using PCR capable of amplifying subgenomic RNA molecules produced during replication (*15*). Our results show that, in laboratories where reverse-transcription PCR is not available but appropriate reagents are available, immunofluorescence testing is a simple and rapid method of assessing whether cells exposed to respiratory or enteric specimens are infected with the virus.

On the basis of this study and earlier reports (*10**,**14**,**15*), monkey kidney cell lines are particularly susceptible to SARS-CoV infection. African green, cynomolgus, and rhesus monkey kidney cell lines have all been previously shown to be susceptible. We identified for the first time that kidney cells derived from a fourth nonhuman primate species, buffalo green monkey, are productively infected with SARS-CoV, with titers that reach 4.7 log_10_/mL above input virus, similar to levels in other monkey kidney cells. We found most monkey kidney–derived cell lines, including BGM, CV-1, FRhK, LLC-Mk2, MA-104, pCMK, and Vero E6, supported replication of SARS-CoV, with titers 3.9–4.7 log_10_/mL above input virus titers. High titers of SARS-CoV attainable in these cell lines should be considered when using them for virus isolation purposes, and appropriate safety guidelines should be followed.

The ability of SARS-CoV to replicate efficiently in kidney-derived cell lines is not surprising given that its functional receptor, the metalloprotease angiotensin-converting enzyme 2 (ACE-2), is highly expressed in kidney tissue (*16*). This metalloprotease receptor is widely divergent from the aminopeptidase N receptor of group 1 CoVs (*16*) but is expressed in lung, heart, kidney, and gastrointestinal tissue, consistent with the pathology of SARS.

Generally, close agreement was seen between our results and those previously reported (*15*), although a difference was seen in CPE. We showed that HEK-293, Huh-7, and pCMK cells supported development of SARS-CoV–specific CPE, whereas no CPE was observed in these cell lines in an earlier study, although replication occurred (*15*). In that study, cells were observed for CPE for only 2 days after infection, whereas in the present study we observed cells for up to 11 days. In Huh-7 and pCMK cell lines, we observed that CPE often developed slowly and affected a population of cells but did not progress (Figure 1). Neither COS nor MEK cells developed SARS-CoV–specific CPE (Figure 1), despite evidence of replication by PCR and immunofluorescence. COS cells are a derivative of the African green monkey kidney fibroblast cell line CV-1, which is highly susceptible to SARS-CoV. The reason for the decreased level of virus production in related COS cells remains to be determined but may be due to a lower level surface expression of the ACE-2 receptor. Nevertheless, the results for these 2 cell lines highlight the unreliability of CPE as a measure of SARS-CoV replication.

Given that primate kidney–derived cell lines are particularly susceptible to infection with SARS-CoV and virus has been isolated from the kidney of an infected human patient (*10*), we suspect that human kidney–derived cell lines might also support SARS-CoV replication. However, until the study by Gillim-Ross et al. (*15*), no human cell lines had been shown to be productively infected by SARS-CoV. We found, in agreement with that study that, HEK-293 and Huh-7 cells were susceptible to infection with the virus. In addition, we identified a third human cell line, Hep G2, derived from a hepatocellular carcinoma, that was also susceptible to infection, although it produced lower levels of virus-specific RNA than HEK-293 and Huh-7 cells. Hep G2 and Huh-7 cell lines are used in research laboratories to study hepatitis B and C viruses, which suggests that cell lines used for research purposes need to be considered carefully for their potential to support SARS-CoV replication, and guidelines must be established to prevent simultaneous work on multiple different viruses within the same laboratory.

This study has shown that SARS-CoV can be isolated in several cell lines commonly used for diagnostic and research purposes and highlights that the virus can achieve high titers in some cell lines, sometimes in the absence of CPE. These findings are particularly relevant to laboratory scientists undertaking virus-isolation procedures on specimens collected from patients with atypical respiratory disease or in research laboratories where the possibility of simultaneously handling more than 1 virus exists.
